# 1-Decyl-6-nitro-1*H*-benzimidazol-2(3*H*)-one

**DOI:** 10.1107/S1600536811041389

**Published:** 2011-10-12

**Authors:** Younes Ouzidan, Youssef Kandri Rodi, El Mokhtar Essassi, Santiago V. Luis, Michael Bolte, Lahcen El Ammari

**Affiliations:** a Laboratoire de Chimie Organique Appliquée, Université Sidi Mohamed Ben Abdallah, Faculté des Sciences et Techniques, Route d’immouzzer, BP 2202 Fès, Morocco; b Laboratoire de Chimie Organique Hétérocyclique URAC21, Faculté des Sciences, Université Mohammed V-Agdal, Avenue Ibn Battouta, BP 1014, Rabat, Morocco; c Departamento de Quimica Inorganica & Organica, E.S.T.C.E., Universitat Jaume I, E-12080 Castellon, Spain; d Institut für Anorganische Chemie, J.W. Goethe-Universität Frankfurt, Max-von-Laue-Strasse 7, 60438 Frankfurt/Main, Germany; e Laboratoire de Chimie du Solide Appliquée, Faculté des Sciences, Université Mohammed V-Agdal, Avenue Ibn Battouta, BP 1014, Rabat, Morocco

## Abstract

The title mol­ecule, C_17_H_25_N_3_O_3_, is built up from fused six- and five-membered rings linked to a –C_10_H_21_ chain. The fused-ring system is essentially planar, the largest deviation from the mean plane being 0.009 (2) Å. The chain is roughly perpendic­ular to this plane, making a dihedral angle of 79.5 (2)°. In the crystal, N—H⋯O hydrogen bonds build infinite chains along [010]. There are channels in the structure containing disordered hexane. The contribution of this solvent to the scattering power was suppressed using the SQUEEZE option in *PLATON* [Spek (2009 ▶). *Acta Cryst.* D**65**, 148–155].

## Related literature

For the pharmacological and biochemical properties of related compounds, see: Gravatt *et al.* (1994 ▶); Horton *et al.* (2003 ▶); Kim *et al.* (1996 ▶); Roth *et al.* (1997 ▶). For related structures, see Ouzidan *et al.* (2011*a* ▶,**b* ▶).* 
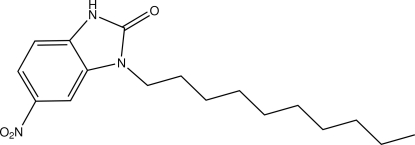


## Experimental

### 

#### Crystal data

C_17_H_25_N_3_O_3_
*M* *_r_* = 319.40
Monoclinic, 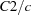

*a* = 32.9827 (6) Å
*b* = 4.55881 (9) Å
*c* = 29.3435 (5) Å
β = 109.481 (2)°
*V* = 4159.56 (13) Å^3^
*Z* = 8
Cu *K*α radiation
μ = 0.57 mm^−1^
*T* = 206 K
0.15 × 0.11 × 0.05 mm

#### Data collection

Agilent SuperNova Dual (Cu at zero) Atlas diffractometer
Absorption correction: analytical [*CrysAlis PRO* (Agilent, 2011) ▶ based on expressions derived by Clark & Reid (1995 ▶)] *T*_min_ = 0.952, *T*_max_ = 0.985
20838 measured reflections
4129 independent reflections
3475 reflections with *I* > 2σ(*I*)
*R*_int_ = 0.029

#### Refinement

*R*[*F* ^2^ > 2σ(*F* ^2^)] = 0.041
*wR*(*F* ^2^) = 0.126
*S* = 1.09
4129 reflections
208 parameters
H-atom parameters constrained
Δρ_max_ = 0.16 e Å^−3^
Δρ_min_ = −0.17 e Å^−3^

### 

Data collection: *CrysAlis PRO* (Agilent, 2011) ▶; cell refinement: *CrysAlis PRO*; data reduction: *CrysAlis PRO*; program(s) used to solve structure: *SHELXS97* (Sheldrick, 2008 ▶); program(s) used to refine structure: *SHELXL97* (Sheldrick, 2008 ▶); molecular graphics: *XP* (Sheldrick, 2008 ▶); software used to prepare material for publication: *SHELXL97*.

## Supplementary Material

Crystal structure: contains datablock(s) I, global. DOI: 10.1107/S1600536811041389/im2324sup1.cif

Structure factors: contains datablock(s) I. DOI: 10.1107/S1600536811041389/im2324Isup2.hkl

Supplementary material file. DOI: 10.1107/S1600536811041389/im2324Isup3.cml

Additional supplementary materials:  crystallographic information; 3D view; checkCIF report

## Figures and Tables

**Table 1:** Hydrogen-bond geometry (Å, °)

*D*—H⋯*A*	*D*—H	H⋯*A*	*D*⋯*A*	*D*—H⋯*A*
N1—H1⋯O1^i^	0.86	1.88	2.743 (1)	178

Symmetry code: (i) 
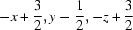
.

**Fig. 1.**
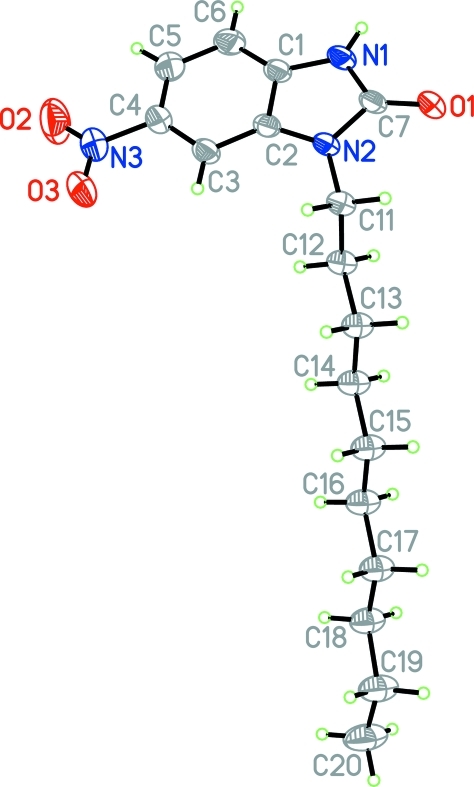
Molecular structure of the title compound with displacement ellipsoids are drawn at the 50% probability level. H atoms are represented as small circles.

**Fig. 2.**
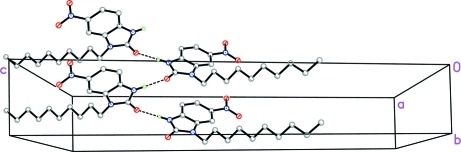
Packing diagram.

## supplementary crystallographic information

### Comment

Benzimidazoles are very useful intermediates/subunits for the development of molecules of pharmaceutical or biological interest. Benzimidazole derivatives have found applications in diverse therapeutic areas including anti-ulcers, anti-hypertensives, anti-virals, anti-fungals and anti-cancers (Gravatt *et al.* 1994; Horton *et al.* 2003; Kim *et al.* 1996; Roth *et al.* 1997).

As a continuation of our research work devoted to the development of substituted benzimidazol-2-one derivatives (Ouzidan *et al.*, 2011*a*, 2011*b*), we report in this paper the synthesis of a new benzimidazol-2-one derivative by action of 1-bromodecane with 5-nitro-1,3-dihydro-benzimidazol-2-one in the presence of a catalytic quantity of tetra-n-butylammonium bromide under mild conditions to furnish the title compound (Scheme 1).

The molecular structure of 1-decyl-6-nitro-1,3-dihydro-benzimidazol-2-one is built up from two fused six-and five-membered rings linked to a C_10_H_21_ chain as schown in Fg.1. The fused rings are essentially planar, with maximum deviations of 0.008 (2) Å and -0.004 (2) Å for C2 and N1, respectively. The dihedral angle between them does not exceed 0.68 (7)°. The torsional angles C7–N2–C11–C12 and C17–C18–C19–C20 are -98.4 (2) ° and 176.7 (2)°, respectively. N1—H···O1 hydrogen bonds build up infinite one-dimensional chains along the [0 1 0] direction as shown in Fig.2 and Table 1.

### Experimental

To 5-nitro-1,3-dihydro-benzimidazol-2-one (0.2 g, 1.1 mmol), potassium carbonate (0.30 g, 2.2 mmol) and tetra-n-butylammonium bromide (0.07 g, 0.2 mmol) in DMF (15 ml) was added 1-bromodecane (0.34 ml, 1.65 mmol). Stirring was continued at room temperature for 6 h. The precipitated salt was removed by filtration and the filtrate was concentrated under reduced pressure. The residue was separated by chromatography on a column of silica gel with ethyl acetate/hexane (1/2) as eluent. Colorless crystals were isolated when the solvent was allowed to evaporate (yield: 27%).

### Refinement

There are channels in the structure containing disordered hexane. The contribution of this solvent to the scattering power was suppressed using the SQUEEZE option in PLATON (Spek, 2009) and the reflections were merged.

H atoms were located in a difference map and treated as riding with C—H = 0.93 Å for all H atoms with *U*_iso_(H) = 1.2 *U*_eq_(aromatic, methine)and *U*_iso_(H) = 1.5 *U*_eq_(methyl).

### Crystal data

**Table:**

C_17_H_25_N_3_O_3_	*F*(000) = 1376
*M**_r_* = 319.40	*D*_x_ = 1.020 Mg m^−^^3^
Monoclinic, *C*2/*c*	Cu *K*α radiation, λ = 1.54184 Å
Hall symbol: -C 2yc	Cell parameters from 8979 reflections
*a* = 32.9827 (6) Å	θ = 2.8–73.1°
*b* = 4.55881 (9) Å	µ = 0.57 mm^−^^1^
*c* = 29.3435 (5) Å	*T* = 206 K
β = 109.481 (2)°	Block, colourless
*V* = 4159.56 (13) Å^3^	0.15 × 0.11 × 0.05 mm
*Z* = 8	

### Data collection

**Table:**

Agilent SuperNova Dual (Cu at zero) Atlas diffractometer	4129 independent reflections
Radiation source: fine-focus sealed tube	3475 reflections with *I* > 2σ(*I*)
mirror	*R*_int_ = 0.029
Detector resolution: 0.4051 pixels mm^-1^	θ_max_ = 73.3°, θ_min_ = 2.8°
ω scans	*h* = −40→40
Absorption correction: analytical [*CrysAlis PRO* (Agilent, 2011) based on expressions derived by Clark & Reid (1995)]	*k* = −4→5
*T*_min_ = 0.952, *T*_max_ = 0.985	*l* = −36→36
20838 measured reflections	

### Refinement

**Table:**

Refinement on *F*^2^	Primary atom site location: structure-invariant direct methods
Least-squares matrix: full	Secondary atom site location: difference Fourier map
*R*[*F*^2^ > 2σ(*F*^2^)] = 0.041	Hydrogen site location: difference Fourier map
*wR*(*F*^2^) = 0.126	H-atom parameters constrained
*S* = 1.09	*w* = 1/[σ^2^(*F*_o_^2^) + (0.0706*P*)^2^ + 0.8537*P*] where *P* = (*F*_o_^2^ + 2*F*_c_^2^)/3
4129 reflections	(Δ/σ)_max_ = 0.001
208 parameters	Δρ_max_ = 0.16 e Å^−^^3^
0 restraints	Δρ_min_ = −0.17 e Å^−^^3^

### Special details

**Table:**

Experimental. CrysAlisPro, Agilent Technologies, Version 1.171.35.11 (release 16-05-2011 CrysAlis171 .NET) Analytical numeric absorption correction using a multifaceted crystal model based on expressions derived by R.C. Clark & J.S. Reid. Clark & Reid (1995).
Geometry. All s.u.'s (except the s.u. in the dihedral angle between two l.s. planes) are estimated using the full covariance matrix. The cell s.u.'s are taken into account individually in the estimation of s.u.'s in distances, angles and torsion angles; correlations between s.u.'s in cell parameters are only used when they are defined by crystal symmetry. An approximate (isotropic) treatment of cell s.u.'s is used for estimating s.u.'s involving l.s. planes.
Refinement. Refinement of *F*^2^ against all reflections. The weighted *R*-factor *wR* and goodness of fit *S* are based on *F*^2^, conventional *R*-factors *R* are based on *F*, with *F* set to zero for negative *F*^2^. The threshold expression of *F*^2^ > σ(*F*^2^) is used only for calculating *R*-factors(gt) *etc*. and is not relevant to the choice of reflections for refinement. *R*-factors based on *F*^2^ are statistically about twice as large as those based on *F*, and *R*- factors based on all data will be even larger.

### Fractional atomic coordinates and isotropic or equivalent isotropic displacement parameters (Å^2^)

**Table:**

	*x*	*y*	*z*	*U*_iso_*/*U*_eq_	
N1	0.76316 (3)	0.5176 (2)	0.70158 (3)	0.0389 (3)	
H1	0.7727	0.4612	0.7312	0.047*	
N2	0.72508 (3)	0.7438 (2)	0.63471 (3)	0.0319 (2)	
N3	0.80707 (4)	0.2956 (3)	0.53894 (4)	0.0493 (3)	
O1	0.70806 (3)	0.8310 (2)	0.70415 (3)	0.0459 (3)	
O2	0.83330 (4)	0.1068 (3)	0.53903 (4)	0.0817 (4)	
O3	0.78870 (3)	0.4442 (2)	0.50339 (3)	0.0571 (3)	
C1	0.77940 (3)	0.4278 (3)	0.66624 (4)	0.0337 (3)	
C2	0.75516 (3)	0.5736 (3)	0.62357 (4)	0.0304 (3)	
C3	0.76366 (3)	0.5367 (3)	0.58100 (4)	0.0341 (3)	
H3	0.7482	0.6341	0.5527	0.041*	
C4	0.79688 (4)	0.3445 (3)	0.58314 (4)	0.0384 (3)	
C5	0.82078 (4)	0.1954 (3)	0.62471 (5)	0.0451 (3)	
H5	0.8425	0.0676	0.6240	0.054*	
C6	0.81208 (4)	0.2379 (3)	0.66730 (4)	0.0433 (3)	
H6	0.8278	0.1413	0.6956	0.052*	
C7	0.72984 (3)	0.7087 (3)	0.68291 (4)	0.0350 (3)	
C11	0.69093 (3)	0.9198 (3)	0.60136 (4)	0.0325 (3)	
H11A	0.7026	1.0276	0.5800	0.039*	
H11B	0.6806	1.0613	0.6197	0.039*	
C12	0.65344 (3)	0.7327 (3)	0.57121 (4)	0.0342 (3)	
H12A	0.6418	0.6247	0.5925	0.041*	
H12B	0.6637	0.5915	0.5529	0.041*	
C13	0.61786 (4)	0.9169 (3)	0.53654 (4)	0.0355 (3)	
H13A	0.6063	1.0490	0.5551	0.043*	
H13B	0.6300	1.0352	0.5168	0.043*	
C14	0.58145 (4)	0.7315 (3)	0.50369 (4)	0.0380 (3)	
H14A	0.5691	0.6155	0.5235	0.046*	
H14B	0.5931	0.5970	0.4856	0.046*	
C15	0.54594 (4)	0.9122 (3)	0.46821 (4)	0.0403 (3)	
H15A	0.5584	1.0310	0.4488	0.048*	
H15B	0.5339	1.0442	0.4863	0.048*	
C16	0.50987 (4)	0.7262 (3)	0.43470 (5)	0.0409 (3)	
H16A	0.5220	0.5927	0.4170	0.049*	
H16B	0.4973	0.6089	0.4541	0.049*	
C17	0.47449 (4)	0.9044 (3)	0.39880 (5)	0.0417 (3)	
H17A	0.4611	1.0287	0.4165	0.050*	
H17B	0.4873	1.0311	0.3808	0.050*	
C18	0.43994 (4)	0.7188 (3)	0.36328 (5)	0.0444 (3)	
H18A	0.4535	0.5884	0.3466	0.053*	
H18B	0.4263	0.5982	0.3812	0.053*	
C19	0.40558 (4)	0.8947 (3)	0.32606 (5)	0.0530 (4)	
H19A	0.3908	1.0171	0.3426	0.064*	
H19B	0.4193	1.0233	0.3092	0.064*	
C20	0.37271 (5)	0.7073 (4)	0.28924 (6)	0.0673 (5)	
H20A	0.3528	0.8315	0.2659	0.101*	
H20B	0.3575	0.5894	0.3053	0.101*	
H20C	0.3871	0.5820	0.2732	0.101*	

### Atomic displacement parameters (Å^2^)

**Table:**

	*U*^11^	*U*^22^	*U*^33^	*U*^12^	*U*^13^	*U*^23^
N1	0.0368 (5)	0.0594 (7)	0.0176 (4)	−0.0018 (5)	0.0051 (3)	0.0043 (4)
N2	0.0304 (4)	0.0432 (6)	0.0192 (4)	−0.0013 (4)	0.0046 (3)	−0.0007 (4)
N3	0.0505 (6)	0.0633 (8)	0.0392 (6)	0.0032 (6)	0.0218 (5)	−0.0011 (5)
O1	0.0411 (4)	0.0714 (7)	0.0241 (4)	−0.0004 (4)	0.0095 (3)	−0.0087 (4)
O2	0.0945 (9)	0.1005 (10)	0.0648 (7)	0.0446 (8)	0.0461 (7)	0.0090 (7)
O3	0.0644 (6)	0.0789 (8)	0.0333 (5)	0.0066 (5)	0.0236 (4)	0.0067 (5)
C1	0.0310 (5)	0.0458 (7)	0.0217 (5)	−0.0058 (5)	0.0051 (4)	0.0020 (4)
C2	0.0287 (5)	0.0372 (6)	0.0228 (5)	−0.0059 (4)	0.0054 (4)	−0.0003 (4)
C3	0.0357 (5)	0.0422 (7)	0.0227 (5)	−0.0028 (5)	0.0075 (4)	0.0027 (4)
C4	0.0390 (6)	0.0479 (7)	0.0299 (6)	−0.0012 (5)	0.0137 (5)	−0.0006 (5)
C5	0.0398 (6)	0.0542 (8)	0.0399 (6)	0.0078 (6)	0.0115 (5)	0.0037 (6)
C6	0.0393 (6)	0.0544 (8)	0.0312 (6)	0.0046 (6)	0.0050 (5)	0.0098 (5)
C7	0.0316 (5)	0.0510 (7)	0.0199 (5)	−0.0082 (5)	0.0052 (4)	−0.0048 (5)
C11	0.0329 (5)	0.0361 (6)	0.0251 (5)	−0.0005 (5)	0.0050 (4)	−0.0008 (4)
C12	0.0330 (6)	0.0345 (6)	0.0299 (5)	−0.0011 (5)	0.0037 (4)	−0.0006 (5)
C13	0.0330 (5)	0.0337 (7)	0.0341 (6)	0.0002 (5)	0.0037 (4)	−0.0001 (5)
C14	0.0349 (6)	0.0345 (7)	0.0366 (6)	0.0005 (5)	0.0013 (5)	−0.0007 (5)
C15	0.0350 (6)	0.0365 (7)	0.0403 (6)	0.0008 (5)	0.0003 (5)	−0.0010 (5)
C16	0.0367 (6)	0.0362 (7)	0.0403 (6)	0.0005 (5)	0.0000 (5)	−0.0011 (5)
C17	0.0366 (6)	0.0381 (7)	0.0413 (6)	0.0013 (5)	0.0006 (5)	−0.0014 (5)
C18	0.0372 (6)	0.0414 (7)	0.0436 (7)	0.0011 (5)	−0.0011 (5)	−0.0024 (5)
C19	0.0447 (7)	0.0489 (9)	0.0499 (7)	0.0060 (6)	−0.0050 (6)	−0.0026 (6)
C20	0.0483 (8)	0.0687 (11)	0.0608 (9)	0.0079 (7)	−0.0140 (7)	−0.0078 (8)

### Geometric parameters (Å, °)

**Table:**

N1—C7	1.3659 (16)	C13—C14	1.5208 (15)
N1—C1	1.3785 (15)	C13—H13A	0.9700
N1—H1	0.8600	C13—H13B	0.9700
N2—C7	1.3793 (13)	C14—C15	1.5235 (15)
N2—C2	1.3819 (15)	C14—H14A	0.9700
N2—C11	1.4612 (14)	C14—H14B	0.9700
N3—O2	1.2198 (16)	C15—C16	1.5235 (16)
N3—O3	1.2218 (15)	C15—H15A	0.9700
N3—C4	1.4612 (15)	C15—H15B	0.9700
O1—C7	1.2304 (14)	C16—C17	1.5201 (16)
C1—C6	1.3745 (18)	C16—H16A	0.9700
C1—C2	1.4077 (15)	C16—H16B	0.9700
C2—C3	1.3789 (14)	C17—C18	1.5194 (16)
C3—C4	1.3879 (17)	C17—H17A	0.9700
C3—H3	0.9300	C17—H17B	0.9700
C4—C5	1.3890 (17)	C18—C19	1.5148 (17)
C5—C6	1.3862 (18)	C18—H18A	0.9700
C5—H5	0.9300	C18—H18B	0.9700
C6—H6	0.9300	C19—C20	1.5138 (19)
C11—C12	1.5197 (15)	C19—H19A	0.9700
C11—H11A	0.9700	C19—H19B	0.9700
C11—H11B	0.9700	C20—H20A	0.9600
C12—C13	1.5234 (15)	C20—H20B	0.9600
C12—H12A	0.9700	C20—H20C	0.9600
C12—H12B	0.9700		
			
C7—N1—C1	110.52 (9)	C14—C13—H13B	109.0
C7—N1—H1	124.7	C12—C13—H13B	109.0
C1—N1—H1	124.7	H13A—C13—H13B	107.8
C7—N2—C2	109.41 (9)	C13—C14—C15	113.41 (10)
C7—N2—C11	123.32 (9)	C13—C14—H14A	108.9
C2—N2—C11	127.13 (8)	C15—C14—H14A	108.9
O2—N3—O3	122.85 (11)	C13—C14—H14B	108.9
O2—N3—C4	118.60 (11)	C15—C14—H14B	108.9
O3—N3—C4	118.55 (11)	H14A—C14—H14B	107.7
C6—C1—N1	131.94 (10)	C14—C15—C16	113.39 (10)
C6—C1—C2	121.76 (10)	C14—C15—H15A	108.9
N1—C1—C2	106.31 (10)	C16—C15—H15A	108.9
C3—C2—N2	131.46 (10)	C14—C15—H15B	108.9
C3—C2—C1	121.41 (10)	C16—C15—H15B	108.9
N2—C2—C1	107.12 (9)	H15A—C15—H15B	107.7
C2—C3—C4	115.61 (10)	C17—C16—C15	113.80 (10)
C2—C3—H3	122.2	C17—C16—H16A	108.8
C4—C3—H3	122.2	C15—C16—H16A	108.8
C3—C4—C5	123.84 (11)	C17—C16—H16B	108.8
C3—C4—N3	117.89 (10)	C15—C16—H16B	108.8
C5—C4—N3	118.28 (12)	H16A—C16—H16B	107.7
C6—C5—C4	119.74 (12)	C18—C17—C16	113.88 (10)
C6—C5—H5	120.1	C18—C17—H17A	108.8
C4—C5—H5	120.1	C16—C17—H17A	108.8
C1—C6—C5	117.64 (11)	C18—C17—H17B	108.8
C1—C6—H6	121.2	C16—C17—H17B	108.8
C5—C6—H6	121.2	H17A—C17—H17B	107.7
O1—C7—N1	127.86 (10)	C19—C18—C17	114.19 (11)
O1—C7—N2	125.50 (11)	C19—C18—H18A	108.7
N1—C7—N2	106.64 (9)	C17—C18—H18A	108.7
N2—C11—C12	112.17 (10)	C19—C18—H18B	108.7
N2—C11—H11A	109.2	C17—C18—H18B	108.7
C12—C11—H11A	109.2	H18A—C18—H18B	107.6
N2—C11—H11B	109.2	C20—C19—C18	113.67 (12)
C12—C11—H11B	109.2	C20—C19—H19A	108.8
H11A—C11—H11B	107.9	C18—C19—H19A	108.8
C11—C12—C13	112.06 (10)	C20—C19—H19B	108.8
C11—C12—H12A	109.2	C18—C19—H19B	108.8
C13—C12—H12A	109.2	H19A—C19—H19B	107.7
C11—C12—H12B	109.2	C19—C20—H20A	109.5
C13—C12—H12B	109.2	C19—C20—H20B	109.5
H12A—C12—H12B	107.9	H20A—C20—H20B	109.5
C14—C13—C12	112.76 (10)	C19—C20—H20C	109.5
C14—C13—H13A	109.0	H20A—C20—H20C	109.5
C12—C13—H13A	109.0	H20B—C20—H20C	109.5

### Hydrogen-bond geometry (Å, °)

**Table:**

*D*—H···*A*	*D*—H	H···*A*	*D*···*A*	*D*—H···*A*
N1—H1···O1^i^	0.86	1.88	2.743 (1)	178.

Symmetry codes: (i) −*x*+3/2, *y*−1/2, −*z*+3/2.
